# Exploring the Healthy Eye Microbiota Niche in a Multicenter Study

**DOI:** 10.3390/ijms231810229

**Published:** 2022-09-06

**Authors:** Davide Borroni, Andreu Paytuví-Gallart, Walter Sanseverino, Carmen Gómez-Huertas, Paola Bonci, Vito Romano, Giuseppe Giannaccare, Miguel Rechichi, Alessandro Meduri, Giovanni William Oliverio, Carlos Rocha-de-Lossada, on behalf of LUCY Consortium

**Affiliations:** 1 Department of Doctoral Studies, Riga Stradins University, LV-1007 Riga, Latvia; 2 Eyemetagenomics Ltd., 71–75, Shelton Street, Covent Garden, London WC2H 9JQ, UK; 3 Sequentia Biotech SL, Carrer del Dr. Trueta, 179, 08005 Barcelona, Spain; 4 Department of Ophthalmology, Hospital Universitario Virgen de las Nieves, 18014 Granada, Spain; 5 Ospedale Civile di Ravenna, Banca Delle Cornee Della Regione Emilia-Romagna, 48121 Ravenna, Italy; 6 Department of Medical and Surgical Specialties, Radiological Specialties and Public Health, 9297 University of Brescia, ASST Spedali Civili, 25100 Brescia, Italy; 7 Department of Ophthalmology, University Magna Graecia of Catanzaro, 88100 Catanzaro, Italy; 8 Centro Polispecialistico Mediterraneo, 88050 Sellia Marina, Italy; 9 Biomedical Science Department, Institute of Ophthalmology, University of Messina, Via Consolare Valeria, 98146 Messina, Italy; 10 Department of Ophthalmology, Qvision (Vithas Almeria), 04120 Almería, Spain; 11 Hospital Regional Universitario de Malaga, 29010 Malaga, Spain; 12 Departamento de Cirugía, Área de Oftalmología, Universidad de Sevilla, 41004 Sevilla, Spain

**Keywords:** microbiome, healthy, metagenomics, eye, 16, OSDI

## Abstract

Purpose: This study aims to explore and characterize healthy eye microbiota. Methods: Healthy subjects older than 18 years were selected for this descriptive cross-sectional study. Samples were collected with an eSwab with 1 mL of Liquid Amies Medium (Copan Brescia, Italy). Following DNA extraction, libraries preparation, and amplification, PCR products were purified and end-repaired for barcode ligation. Libraries were pooled to a final concentration of 26 pM. Template preparation was performed with Ion Chef according to Ion 510, Ion 520, and Ion 530 Kit-Chef protocol. Sequencing of the amplicon libraries was carried out on a 520 or 530 chip using the Ion Torrent S5 system (Thermo Fisher; Waltham, MA, USA). Raw reads were analyzed with GAIA (v 2.02). Results: Healthy eye microbiota is a low-diversity microbiome. The vast majority of the 137 analyzed samples were highly enriched with *Staphylococcus*, whereas only in a few of them, other genera such as *Bacillus, Pseudomonas,* and *Corynebacterium* predominate. We found an average of 88 genera with an average Shannon index of 0.65. Conclusion: We identified nine different ECSTs. A better understanding of healthy eye microbiota has the potential to improve disease diagnosis and personalized regimens to promote health.

## 1. Introduction

The Ocular Microbiome Project started in 2010 exploring the potential physiopathological relationship between the ocular microbiome and microbiota and ocular diseases [1]. The term microbiome refers to the genetic material of bacteria, fungi, viruses, protozoa, and eukaryotes that are found in a specific tissue, while the term microbiota refers to the community of microorganisms that is present in that specific tissue [2]. It is now clear that ocular microbiota includes all types of microorganisms, both commensal and pathogenic, living on or in the eye. It can regulate, defend, or provoke and perpetuate the development of chronic eye diseases [3,4]. 

The composition of the microbiome may vary depending on the methods of analysis. Microbial culture and smears remain the gold standard for diagnosis of ocular infections although the low yield, their low sensitivity, the inability to detect some organisms, and the long time needed to have the results are important limits of the method [5]. To overcome the limitations of traditional analysis, DNA-based molecular diagnostic techniques, able to detect specific nucleic acid sequences, are an emerging area of research known as metagenomics [6]. 

Metagenomics is a new discipline in genomic analysis, based on the development of next-generation sequencing (NGS) technology, able to detect all microorganisms and their genetic material included in a biological sample [7]. It is even more becoming a useful tool also in clinical practice, as it may improve diagnostic yield. Likewise, it is unbiased, a hypothesis-free approach, and it is shown to be useful for functional as well as taxonomic profiling [8,9,10,11,12,13]. These newer molecular metagenomics methods allow researchers to expand the previously reported knowledge of ocular surface (OS) microbial diversity [14].

Recent metagenomic studies suggest that healthy OS is characterized by a relatively stable microbiota with low diversity. A “core” microbiota shares a few taxa in all individuals [15], including commensal, environmental, and opportunistic pathogenic bacteria [16]. *Proteobacteria*, *Actinobacteria,* and *Firmicutes* are the dominant phyla on the OS. The most common taxa at the genus level are *Pseudomonas*, *Propionibacterium*, *Bradyrhizobium, Corynebacterium, Acinetobacter*, *Brevundimonas, Staphylococci*, *Aquabacterium*, *Sphingomonas*, *Streptococcus*, *Streptophyta,* and *Methylobacterium*, although potential contamination may have influenced their identification [17,18,19,20,21,22,23,24,25,26].

Even with this great diversity, the OS microbiota remains much less rich in comparison to those of the gut, skin, or oral cavity [27,28,29,30,31].

Aside from this, stratification of the ocular microbiome has not yet been described in healthy subjects so far. The concept of enterotype was described in 2011 by Arumugam et al., who established the idea that the gut microbiome could be stratified into three different clusters of bacterial communities: gut microbiomes rich in *Bacteroides*, rich in *Prevotella*, and rich in *Ruminococcus* [32]. Likewise, the equivalent term in the vaginal microbiome is “community state type” or CST. There are five CSTs: CST I, II, III, and V are dominated by *L. crispatus*, *L. gasseri*, *L. iners*, and *L. jensenii*, respectively, while CST IV is dominated by other species different than *Lactobacillus*.

We propose the term eye community state type (ECST) as a concept for stratifying the different profiles of bacterial communities that coexist together in a healthy eye.

Therefore, it is clear that understanding the composition and function of a normal ocular microbiome and/or microbiota represents a critical starting point for a targeting therapy and possibly the development of adequate probiotic products, which could improve homeostasis and the imbalance (dysbiosis) caused by certain diseases [26].

The aim of the study is to use the 16S rRNA sequencing method to explore the healthy eye microbiota with the highest sample and set the eye microbiome parameters for future comparative studies.

## 2. Results

In total, 137 samples were collected from 137 patients, aged between 18 and 82 years. Of those, 53 were males, and 84 were females. In terms of location, 69 were collected in northern Italy (Bologna), 48 in southern Spain—Virgen de las Nieves University Hospital (Granada, Spain), and 20 in southern Italy—Sellia Marina (Italy).

**Figure 1:**
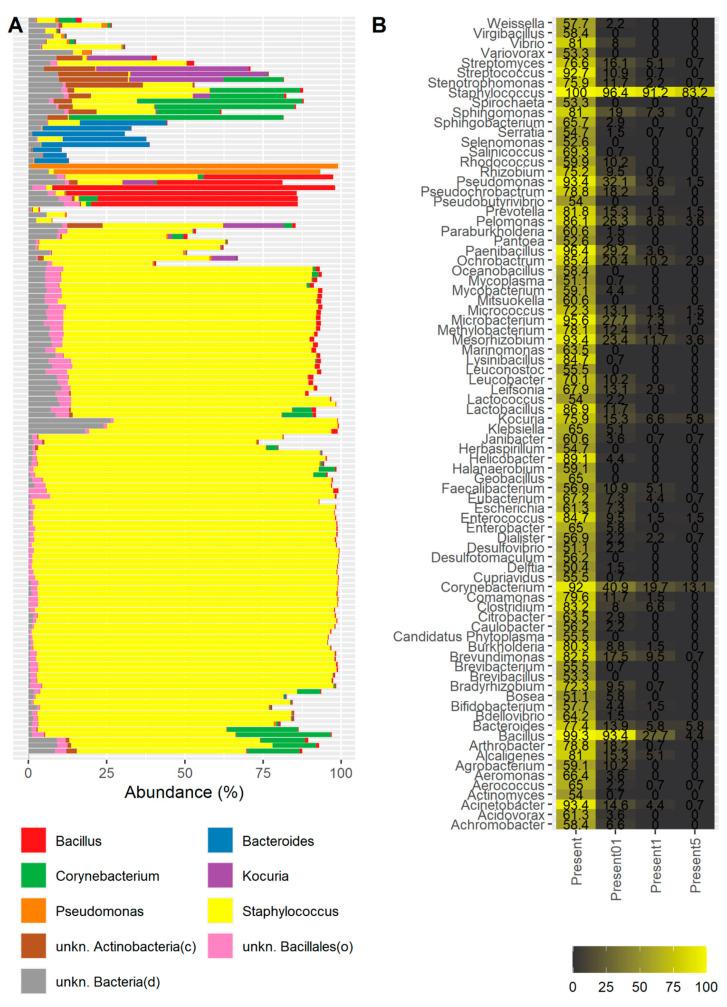
Taxonomic profiling of the samples: (**A**) bar plot showing the taxonomic composition of the genera above an average abundance of 1% across all samples; (**B**) heatmap showing the most prevalent genera across samples. Only those genera present in at least 50% of the samples are shown (1st column). The 2nd, 3rd, and 4th columns show the prevalence of the corresponding genera considering a minimum abundance of 0.1%, 1%, and 5%, respectively.

The most abundant genera were *Staphylococcus* (60.88%, on average across all samples), followed by *Bacillus* (3.3%, on average), and *Corynebacterium* (3.12%, on average) (Figure 1A), although in some samples, other genera such as *Pseudomonas*, *Kocuria*, *Aerococcus*, or *Chryseobacterium* predominated (Figure 1A, Supplementary Figures S1 and S2).

We also assessed the prevalence of the genera under study across the samples. Under the criterion that a genus is identified in a sample if it has a support of one read or more, we observed that *Staphylococcus* was present in all the samples from our cohort (Figure 1B). The genus *Bacillus* was also identified in almost all the samples, with a prevalence of 99.3%. Other genera that were highly prevalent were *Paenibacillus* (96.35%), *Microbacterium* (95.62%), *Mesorhizobium* (93.43%), *Acinetobacter* (93.43%), *Pseudomonas* (93.43%), and *Streptococcus* (92.7%).

The prevalence of highly distributed genera sharply dropped when another criterion was considered: Under this condition, a genus is considered to be present in a sample if its relative abundance is at least 0.1%. In this case, *Staphylococcus* and *Bacillus* still maintained a high prevalence of 96.4% and 93.4%, respectively. The third most prevalent genus was *Corynebacterium* (40.87%). Considering more stringent cutoffs to evaluate a genus to be present in a sample, *Staphylococcus* was present with a minimum abundance of 1% in 91.2% of the samples and with a minimum abundance of 5% in 83.2% of the samples. Additionally, *Bacillus* was present with a minimum abundance of 1% in 27.7% of the samples and with a minimum abundance of 5% in only 4.4% of the samples. *Corynebacterium* maintained a prevalence of 13.1% with at least a relative abundance of 5%.

**Figure 2:**
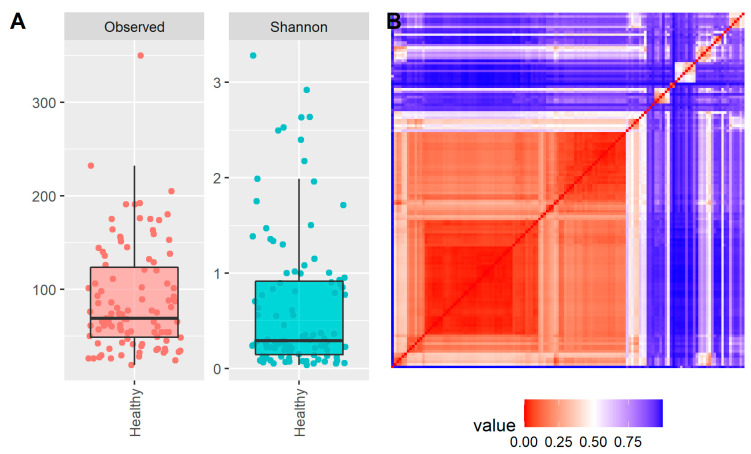
Diversity metrics: (**A**) box plots showing the alpha diversity (observed genera/richness and Shannon) of the samples in our cohort; (**B**) heatmap showing the similarity among the samples using the beta diversity Bray–Curtis dissimilarity metric. Each row and column is related to a sample; the diagonal (from bottom left to top right) shows the same sample comparison.

Alpha diversity indices (richness and Shannon indices) were assessed in the samples of our cohort. In order to perform a proper comparison, samples were rarefied down to the number of reads of the sample located in the first quartile (22.309 reads), thus considering 75% of the samples for the alpha diversity analysis and discarding 25% of the samples with the lowest number of reads. In terms of richness (how many genera are found in a sample), the healthy eye microbiota was found to have an average number of 88 genera and a median of 68 genera (Figure 2A).

The Shannon alpha diversity index accounts for both richness and evenness (how evenly the genera are distributed) in a given sample. In this context, the microbiota of the healthy eye was found to have an average Shannon index of 0.65 and a median of 0.29 (Figure 2A).

The Bray–Curtis beta diversity metric provides a way to assess the similarity or dissimilarity between samples, with values in a range between 0 and 1. A value of 0 suggests two identical samples, while a value of 1 suggests two completely different samples without any genera in common. A pairwise comparative study was performed between all the samples (Figure 2B).

The vast majority of the samples showed high similarity (low beta diversity value) due to the taxonomic profiles of the samples being alike, especially due to the presence of predominant species such as *Staphylococcus*. Nevertheless, small groups of samples showed high similarities between them but low similarities between their group and the rest of the samples, indicative of an independent taxonomic profile.

For samples with alternative taxonomic profiles enriched with other genera, we also performed a clustering analysis that resembles the concept of “enterotyping” described for the gut microbiome, which is defined as a classification of living organisms based on the taxonomic composition of the microbiota (Figure 1A and Figure 2B). For instance, the gut microbiota is defined by three enterotypes depending on the abundance of *Bacteroides*, *Prevotella,* and *Ruminococcus* (Arumugam et al. 2011). In this context, for the case of the healthy eye, we described the different taxonomic profiles found in the healthy eye under the concept of eye community state type (ECST).

**Figure 3:**
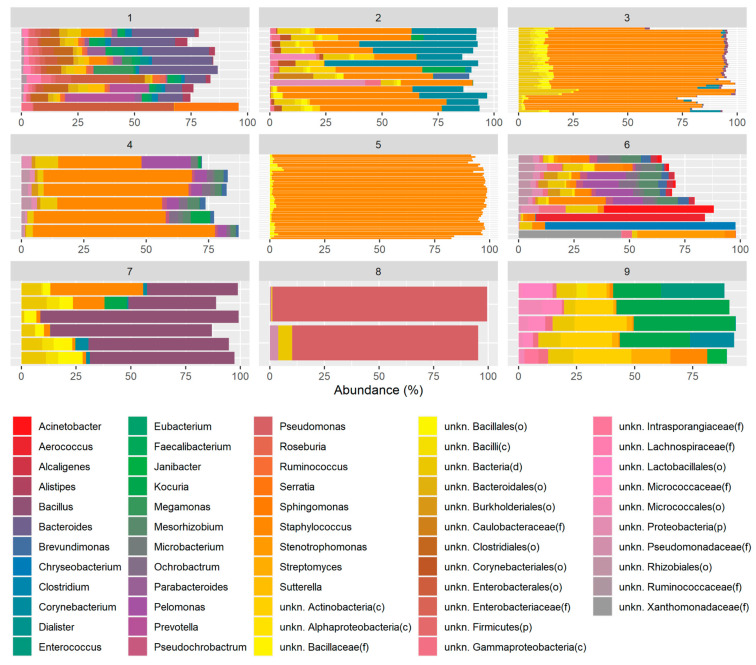
Bar plots showing the taxonomic composition in each enterotype. Only the genera above an average abundance of 1% for each sample enterotype are shown.

During the identification of ECSTs in the healthy eye, we determined that the optimal number of clusters or ECSTs found in the healthy eye microbiota is nine. A total of 9 samples were found in enterotype 1, 13 samples in enterotype 2, 14 samples in enterotype 3, 6 samples in enterotype 4, 46 samples in enterotype 5, 10 samples in enterotype 6, 6 samples in enterotype 7, 2 samples in enterotype 8, and 5 samples in enterotype 9 (Figure 3). About 63% of the samples belonged to clusters 3 and 5.

**Figure 4:**
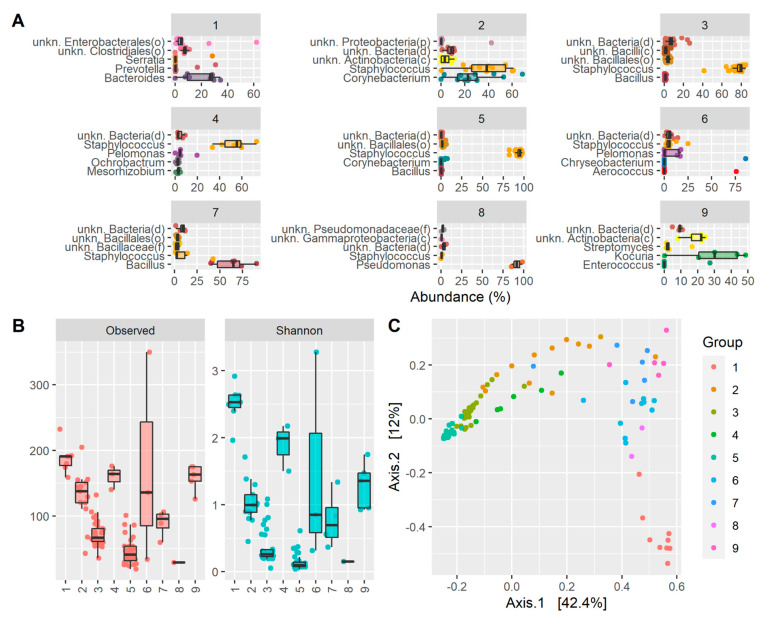
Drivers of ECST classification and diversity metrics: (**A**) box plots showing the top five most abundant genera in each ECST; (**B**) box plots showing the alpha diversity (observed genera and Shannon) for each ECST; (**C**) principal coordinate analysis (PCoA) from Bray-Curtis beta diversity values colored via eECST.

Assessing the taxonomy of each enterotype independently, it was observed that enterotype 1 was enriched with *Bacteroides*, enterotype 2 was high in *Staphylococcus* and *Corynebacterium*, enterotype 3 had a high abundance of *Staphylococcus* and bacteria under the order Bacillales, enterotype 4 had a high abundance of *Staphylococcus* and other different bacteria such as *Pelomonas*, *Ochrobactrum*, or *Mesorhizobium*, enterotype 5 was essentially predominated by *Staphylococcus*, enterotype 6 was high in *Pelomonas* and *Staphylococcus* in less abundance, enterotype 7 was high in *Bacillus* and a small number of *Staphylococcus*, enterotype 8 was very high in *Pseudomonas*, and enterotype 9 was enriched in *Kocuria* (Figure 3 and Figure 4A). In addition, as expected, samples belonging to the same enterotype clustered together in the principal coordinate analysis (PCoA), indicative of a similar taxonomic profile (Figure 4C).

Each ECST is defined by a different set of microorganisms; thus, it is also defined by different alpha diversity metrics. ECSTs 3, 5, and 8 were the ECSTs with the lowest richness and the lowest Shannon index. These ECSTs had *Staphylococcus* (ECSTs 3 and 5) and *Pseudomonas* (ECST 8) as predominant species (Figure 4B). Instead, other ECSTs such as 1, 4, or 9 had a higher number of genera and also a higher Shannon index due to the presence of multiple genera with more even abundances.

## 3. Discussion

Several studies have evaluated the ocular surface microbiome and/or microbiota by using traditional microbiology methods [33,34], but the current knowledge is still growing thanks to the complex analyses performed by genomic technology [19,35]. The metagenomic analysis permits the characterization of the entire microbial community present in a sample as well as the definition of the richness and evenness of this community, although the method does not discriminate between viable and non-viable microbes.

At the genus level, according to the 16S rRNA sequencing studies, *Corynebacterium*, *Acinetobacter*, *Pseudomonas, Staphylococcus*, *Propionibacterium*, and *Streptococcus* have been consistently detected, with variances in the relative abundance, in most of them [4]. Moreover, *Pseudomonas* and *Acinetobacter* have been detected throughout geographic regions by most studies, although with differences in their relative abundance [19]. Although recent analysis has shown a potential diversity of the ocular surface microbiota that includes both pathogenic and non-pathogenic bacteria [16,27,36], overall, the ocular surface appears to be characterized by a relatively constant, paucibacterial microbiota [37]. However, with the exception of the study by Zhou et al., who sampled 105 healthy Gambian volunteers living in a trachoma-endemic community, most studies either have low sample sizes or are reviews attempting to characterize the OS based on the data extrapolated from other studies [38].

In our study, the vast majority of the samples were highly enriched with *Staphylococcus*, whereas in only a few of them, it was not the most abundant genus, and other genera such as *Bacillus*, *Pseudomonas,* and *Corynebacterium* predominated.

Moreover, *Staphylococcus* and *Bacillus* genera, followed by *Corynebacterium*, maintained high levels of prevalence in all the samples considering a minimum cutoff of 0.1%.

In terms of richness and evenness of the genera, evaluated by using the Shannon alpha diversity indices, we found that the microbiota of the healthy eye was characterized by an average number of 88 genera and a median of 68 genera, and an average Shannon index of 0.65 and a median of 0.29. These values are very low in comparison with other human microbiota such as those of the gut, oral cavity, or skin [39], and comparable with the values registered for the vaginal microbiota, which is mainly predominated by the genus *Lactobacillus* [40]. Therefore, these data suggest that the healthy eye microbiota is a low-diversity microbiome, with just a few predominant genera, thus decreasing the evenness of the microbiota such as *Staphylococcus*. Furthermore, our data confirm what is fairly well-established in the literature concerning the Shannon diversity index of the conjunctiva of healthy adults (0.65 average), which is much lower than that exhibited in children, showing a value of 2.3 and, irrespective of this fact, it is lower than those of the eyelid margin (3.4) and periocular skin (3.5) [41].

Most of the samples showed high similarity related to a similar taxonomic profile, although some groups of these samples showed low similarity to each other attesting to a different taxonomic profile but always enriched with *Staphylococcus*. This finding is in accordance with the majority of studies that registered the *Staphylococcus* genus as the most prevalent, although with different levels of abundance between them [15,27,36,37,42].

**Identification of different Eye Community State Types:**

Moreover, although the vast majority of the samples showed a similar taxonomic profile highly enriched with *Staphylococcus*, there were other samples showing alternative taxonomic profiles. Therefore, by performing a clustering analysis that resembles the concept of “enterotyping” in the gut microbiome, described in this study as a process of classification called eye community state types (ECSTs), we were able to describe nine different clusters or ECSTs belonging to the ocular surface microbiome of a healthy eye.

Even with this kind of analysis, assessing the taxonomy of each ECST independently, we observed that *Staphylococcus* was present in almost all the ECSTs (2, 3, 4, 5, 6, and 7), although it was most predominant in ECSTs 3 and 5, characterized by the lowest alpha diversity index. ECSTs 1, 8, and 9 instead were enriched with other genera such as *Bacteroides, Pseudomonas,* and *Kocuria*, respectively, with *Pseudomonas* predominant in ECST 8, which indeed showed a low alpha diversity due to its highest prevalence. In contrast, ECSTs 1, 4, and 9 showed a higher Shannon index due to the presence of multiple genera with more even abundances.

However, in terms of the genus, although greater alpha diversity was found in some of the ECSTs analyzed, it was observed only and exclusively in those clusters that were not dominated by *Staphylococcus*, confirming that it is the predominant genus in the OS microbiota of a healthy human eye.

In the present study, the difference between inferior and superior conjunctiva was not analyzed [43]. In addition, a characterization of the ocular microbiota should also consider stratification by sex, age, and other potential confounders. Regarding the influence of gender on the ocular surface microbiome, it is still debated, and most publications do not mention any difference [19,37,38,44,45,46], while others showed a difference in abundance of certain genera or Shannon diversity [15,27]. Other limitations include the fact that this was a multicenter study with the participation of different investigators. Although all of them are experienced ophthalmologists specialized in the OS, and all were instructed on how to take the samples, we cannot guarantee that all the samples were free from contamination. Likewise, we did not analyze the virome or fungal microbiome of OS using the 18S or shotgun analysis. We plan to perform this analysis in future research. Likewise, in this first study, we did not study the genetic material of the microbiota. We also plan to conduct this analysis in the future. Nonetheless, we believe that our study helps to consolidate the knowledge about the composition of the healthy ocular surface microbiota in a large sample and, for the first time, shows the valuable results of the metagenomic-based “enterotyping” process in the healthy eye, defined as the ECST.

## 4. Materials and Methods

### 4.1. Recruitment of Subjects

This study was approved by the institutional review board of the Riga Stradins University nr.29/20092016. Patients visited the following ophthalmology clinics: Virgen de las Nieves University Hospital (Granada, Spain), Emilia-Romagna Eye Bank (Bologna, Italy), and Centro Polispecialistico Mediterraneo (Sellia Marina, Italy). This descriptive cross-sectional study followed the tenets of the Declaration of Helsinki, and informed consent was obtained from human subjects involved in the study. Healthy subjects older than 18 years were selected for the study. Recruitment was based on the inclusion/exclusion criteria and on the Ocular Surface Disease Index (OSDI) score.

Healthy subjects were defined as subjects who had no ocular surgery, allergy or type of ocular inflammation, use of contact lens, ocular surface diseases, meibomian gland dysfunction, did not take any antibiotic doses in the past 6 months, and did not take any tablet for systemic diseases (since this fact could change the ocular microbiota), a BMI index between 18.5 and 24.9, and no hyperglycemia blood levels [26]. All those patients who were considered healthy and met the requirements were invited to participate in the study. Informed consent was obtained from all individuals at the beginning of the study.

### 4.2. Sampling Technique, DNA Extraction, PCR Amplification, Library Preparation, and Amplicon Sequencing

Samples were collected with an eSwab with 1 mL of Liquid Amies Medium (Copan Brescia, Italy). The eSwab was applied on the inferior surface of the eye and moved 2 times “limbus to fornix to limbus”. No fluorescein and anesthetics were used to avoid influences on the eye microbiota [27,47].

DNA was extracted with a QIAamp DNA Microbiome Kit (QIAGEN, Hilden, Germany) in accordance with the manufacturer’s instructions. Extracted DNA was quantified with Agilent TapeStation 4150 using Genomic DNA ScreenTape (Agilent Technologies, Santa Clara, CA, USA). DNA libraries were prepared with an Ion 16S Metagenomics Kit (Thermo Fisher, Waltham, MA, USA). Briefly, bacterial genomic DNA was diluted to 2 ng/mL, and then 2 mL was used for library preparation. Amplification was performed using 2 primer pools containing a primer to amplify the V2-4-8 region of 16S rDNA and the V3-6, 7–9 region of 16S rDNA. After amplification, PCR products were purified and end-repaired for barcode ligation. The last step was library amplification and pooling for template preparation. Libraries were pooled to obtain the final concentration at 26 pM. Template preparation was performed with Ion Chef according to the Ion 510, Ion 520, and Ion 530 Kit-Chef protocol.

Sequencing of the amplicon libraries was carried out on a 520 or 530 chip using the Ion Torrent S5 system (Thermo Fisher, Waltham, MA, USA) according to the supplier’s instructions. After sequencing, the individual sequence reads were filtered using the Ion software to remove low-quality and polyclonal sequences. The sequences matching the IonXpress adaptor were also automatically trimmed. All the S5 quality-approved, trimmed, and filtered data were exported as bam files.

### 4.3. Data Analysis

Raw reads were analyzed with GAIA (v 2.02) (https://metagenomics.sequentiabiotech.com, accessed on 30 August 2022) to obtain operational taxonomic unit (OTU) tables at different taxonomic levels. Gaia is an online easy-to-use proprietary platform for microbiome analyses with a high prediction of 0.978 at the genus level [48]. The present study focused on genera. With the aim to filter putative false positives, only those genera supported by at least 2 reads in at least 2 samples were considered for the downstream analyses. The richness and Shannon alpha diversity metrics, as well as Bray–Curtis beta diversity values, were computed with the R package phyloseq [49]. The identification of ECSTs was carried out with the R package cluster using the PAM clustering algorithm on Bray–Curtis dissimilarity values; the optimal number of enterotypes was obtained using the Gap statistic. Principal coordinate analysis (PCoA) was performed using the function plot_ordination from the R package phyloseq.

### 4.4. Statistical Analysis

Statistical analysis was performed using the R programming language (version 4.0.3) together with Integrated Development Environment (IDE) Rstudio (version 1.1.45).

## 5. Conclusions

The healthy microbiota of the eye is divided into nine different ECSTs. The understanding of the presence of more ECSTs could provide future guidance in medical treatments. ECSTs 3 and 5 were the most predominant ones, accounting for 63% of the samples, and were characterized by a high abundance of *Staphylococcus* together with low alpha diversity. Nevertheless, there were other less frequent healthy microbiotas with other genera and higher alpha diversity. For instance, ECSTs 2 and 4 were also characterized by a high presence of *Staphylococcus*, but they presented higher alpha diversity values with a remarkable presence of other genera (e.g., *Corynebacterium* or *Pelomonas*). With the exception of ECST 8, which showed a low diversity due to the extremely high presence of *Pseudomonas*, the other ECSTs showed alpha diversity values higher than the clusters dominated by *Staphylococcus*.

## Data Availability

Not applicable.

## Supplementary Materials

The supporting information can be downloaded at: https://www.mdpi.com/article/10.3390/ijms231810229/s1.
