# Supplementary material for: Exploring the Healthy Eye Microbiota Niche in a Multicenter Study
Source: Int J Mol Sci. 2022 Sep 6;23(18):10229. doi: 10.3390/ijms231810229 (PMC9499403; doi:10.3390/ijms231810229)
Supplement: Supplementary file 1 [file ijms-23-10229-s001.zip › ijms-1889208-supplementary.pdf]

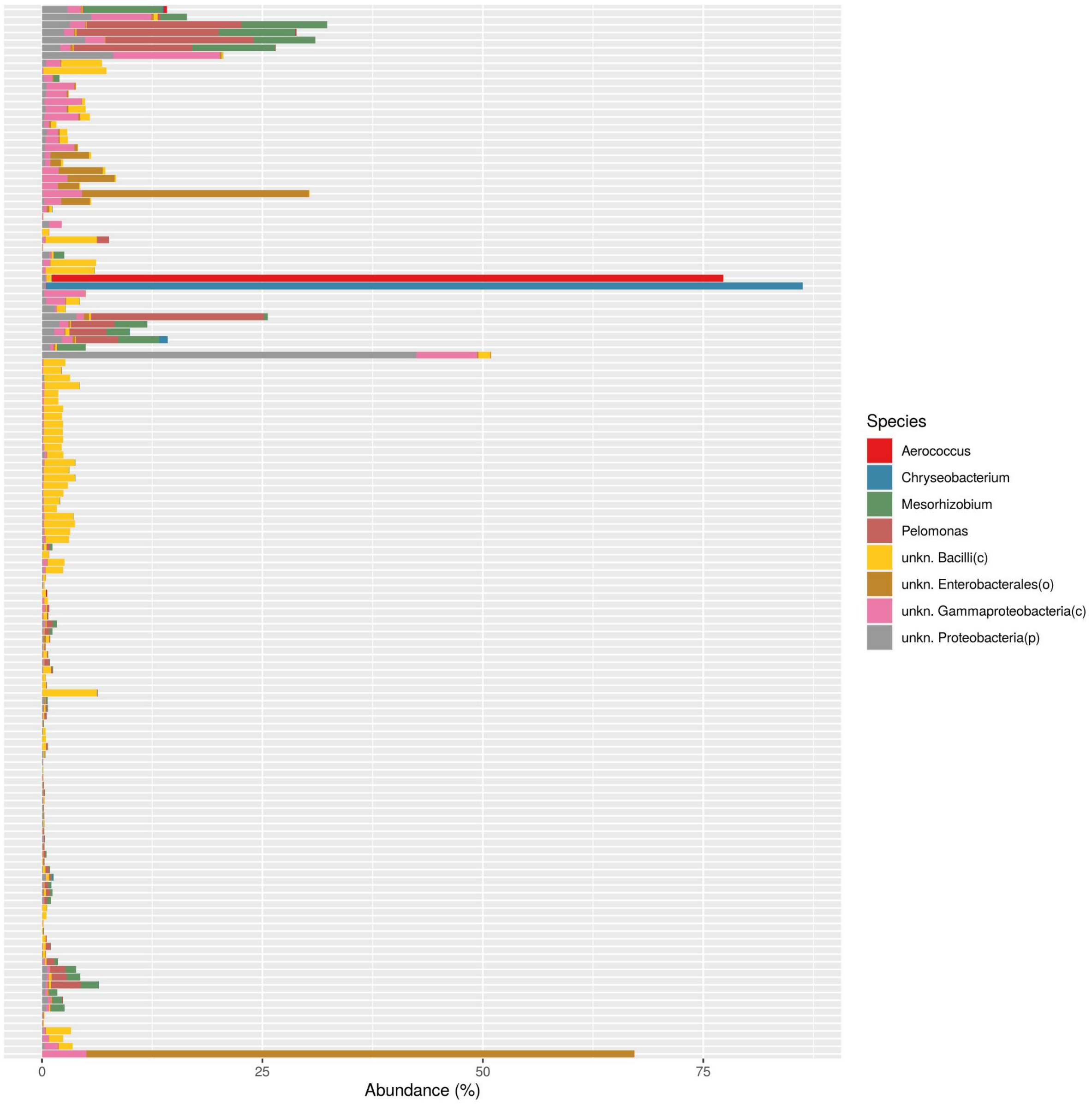

Figure S1. Barplot showing the taxonomic composition of the genera between an average abundance between 0.5-1% across samples.

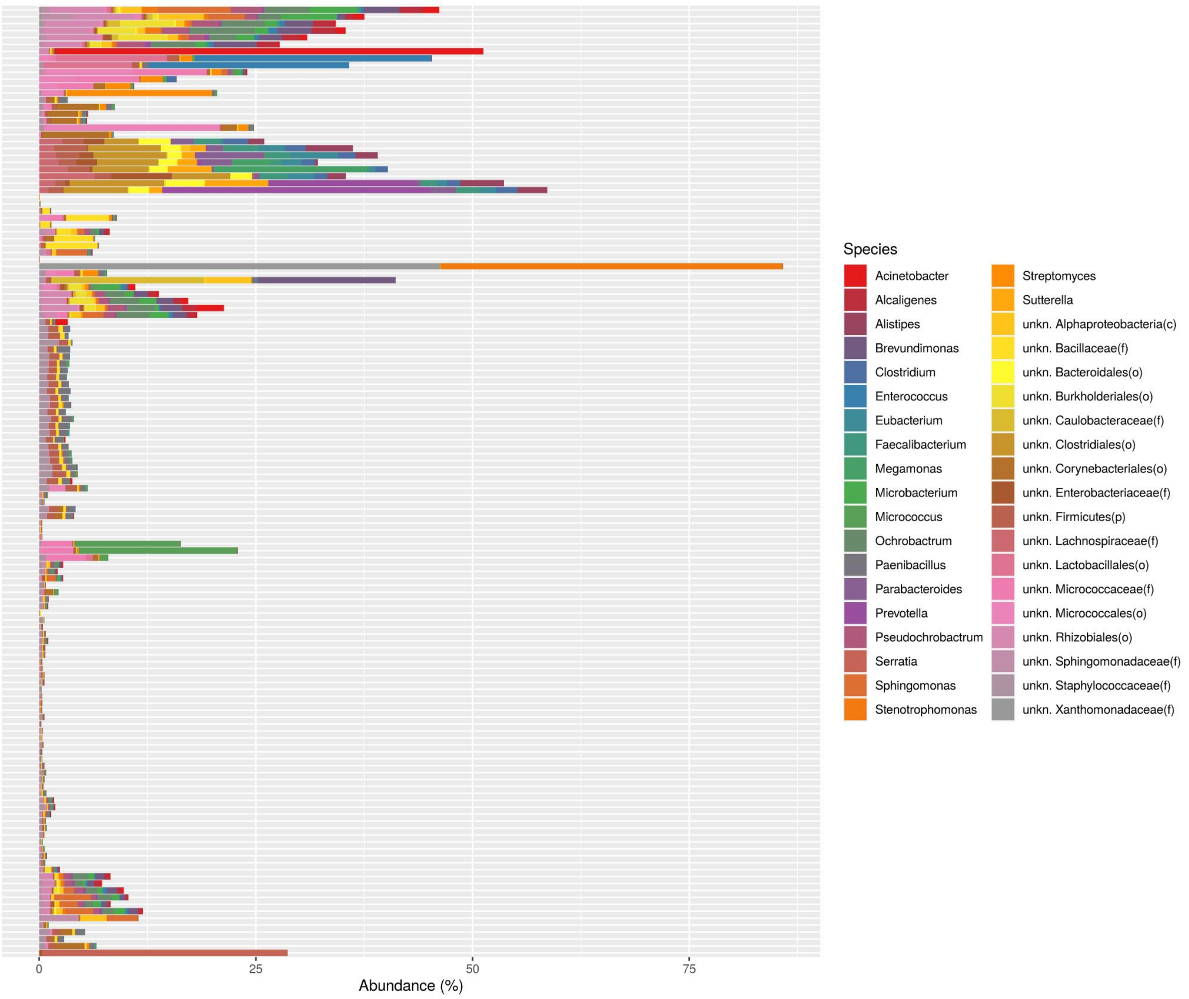

Figure S2. Barplot showing the taxonomic composition of the genera between an average abundance between 0.1-0.5% across samples.
